# Investigating the Matrix of Factor V Leiden (G1691A), Factor II Prothrombin (G2021A), MTHFR C677T and A1298G Polymorphisms in Greek Population: A Preliminary Study

**DOI:** 10.3390/medsci12040061

**Published:** 2024-11-05

**Authors:** Maria Spanoudaki, Aikaterini Itziou, Antonios Cheimaras, Orestis Tsiripidis, Grigoris Risvas, Naysika Tsitlakidou, Vasileios Balis

**Affiliations:** 1 Department of Dietetics, School of Health Sciences, International Hellenic University, 57400 Thessaloniki, Greece; maryspan1@gmail.com (M.S.); antonischeim@gmail.com (A.C.); 2 Clinical Dietetics and Nutritional Department, 424 General Military Hospital, 56429 Thessalonki, Greece; 3 Dietetetics and Biomedical Department, School of Health Sciences Aegean College, 45 Tsimiski Str., 54623 Thessaloniki, Greece; naysikatsitlakidoy86@gmail.com; 4 Department of Midwifery, School of Health Sciences, University of Western Macedonia, 50200 Ptolemaida, Greece; aitziou@uowm.gr; 5 Surgical Department, Bodosakio Hospital, 50200 Ptolemaida, Greece; tsiripore72@gmail.com; 6 Dietetics Department, School of Sciences, Aegean College, 15 Panepistimiou Str., 10564 Athens, Greece; g.risvas@aegeancollege.gr; 7 Quality Management, Regenerative Medicine Centre, Medical School, Aristotle University, 54124 Thessaloniki, Greece

**Keywords:** factor V Leiden, prothrombin G20210A, MTHFR C677T, MTHFR A1298C, cancer, thrombophilia

## Abstract

Background: Thrombophilia, characterized by an increased risk of thrombosis, can result from genetic polymorphisms in clotting factors. This study aims to investigate the prevalence of factor V Leiden (G1691A), factor II prothrombin (G20210A), and MTHFR (C677T and A1298C) polymorphisms in a Greek population, evaluating not only their association with thrombophilia, but also broader health implications. Methods: We conducted a cross-sectional study involving one hundred apparently healthy adults from Thessaloniki, Greece. After obtaining informed consent, DNA was isolated and analyzed using real-time PCR to detect the frequencies of the aforementioned polymorphisms. Results: The genetic distribution of the examined polymorphisms aligns closely with that observed in Northern Europe. Factor V Leiden (FVL) and prothrombin G20210A mutations were predominantly wild types, with a small percentage showing heterozygous mutations. The MTHFR C677T and A1298C polymorphisms showed a higher variation in allele frequency. Certain lifestyle factors such as smoking and high body mass index were significantly associated with the occurrence of combined MTHFR genotypes, suggesting an interaction between genetic and environmental risk factors. Family cancer and cardiovascular history was significantly associated with combined FVL and prothrombin G20210A and MTHFR polymorphism heterozygous carriers. Conclusions: Our findings indicate that these genetic polymorphisms are not only pivotal in understanding thrombophilia but also have broader implications for cardiovascular disease and cancer. This study highlights the need for further research into the combined effects of genetic and epigenetic factors on health, which could lead to improved screening and personalized preventive healthcare strategies.

## 1. Introduction

Thrombophilia can be defined as a predisposition to form clots inappropriately. It can be inherited or acquired, and it is the main factor promoting venous and arterial thromboembolism (VTE). VTE is a disorder of great importance to the health of general population [1].

Thrombosis is a hypercoagulable condition and only the mutual effects between the environment and genes can lead to the possible development of clinical symptoms and signs. Thrombosis mostly occurss in the lower limbs and pulmonary vessels, often leading to pulmonary hypertension, increasing mortality and morbidity, especially in young adults [2].

The presence of specific DNA mutations/polymorphisms in various genes encoding blood clotting factors has been associated with the risk of developing thrombosis, which include genetic variants that predispose to thrombophilia. For example, variants in the prothrombin factor V Leiden (FV) and FII 20210G>A genes lead to increased blood clotting. Increased homocysteine levels can result from several mutations in the methylene-tetrahydro-folate reductase (MTHFR) gene, have been implicated as risk factors for thrombosis, and are involved in peripheral arterial disease [2,3]. In addition, the main types of hereditary thrombophilia are the results of the G1691A mutation of factor V Leiden, which can be homozygous or heterozygous. It has been well established that both homozygous or heterozygous mutations are independent risk factors for thromboembolism and deep vein thrombosis during pregnancy, as well as for recurrent miscarriages [4,5,6]. Inherited thrombophilic disorders are much more widespread than first expected and it is not uncommon to find individuals or families with more than one gene mutation [7].

Epidemiological research clearly has shown that the range of inherited variants of thrombophilia and subsequent clinical features differ among ethnic groups, regions, and between genders [8]. Factor V Leiden G1691A (FVL), prothrombin G2021A (PT20210A), and C677T methylene-tetrahydro-folate reductase (MTHFR C577T), are the most frequently studied polymorphic variants in the last decades as possible genetic risk factors for VTE. It is evident that FVL and PT20210A alone represent important risk factors for VTE. In the meta-analyses of Gohil and colleagues, the heterozygosity for FVL significantly elevated the possibility of a VTE episode by nine and a half fold, while PT20210A by three-fold compared to the wild-type [9]. With regard to C677T MTHFR, the risk of VTE was significantly elevated by almost 60% in Chinese homozygotes compared wild-type homozygotes to heterozygotes, while in Caucasians, no significant correlation was detected [10].

It is generally accepted that specific polymorphic alleles are associated with venous thrombosis and could also provide a moderate risk for arterial thrombosis [9]. For example, clotting factor V (F5) Leiden (G1691A), coagulation factor II (F2) G20210A, 5,10-methylene tetrahydro-folate reductase (MTHFR), C677T, and F2 G20210A are identified in Caucasian people with a frequency between 1–2.5% and 2–4%. On the contrary, MTHFR C677T is widely distributed with a frequency of variant alleles within the range of 5–60%. These gene polymorphisms, alone or in combination, were recently studied in the Greek population living in Athens, revealing differences or similarities with previously published data on other geographical areas [11].

On the other hand, MTR (methionine synthase) and MTHFR (5,10-methylene tetrahydro-folate reductase) are enzymes playing a dominant role in folic acid metabolism [12,13]. MTHFR has a pivotal role in preserving homeostasis between DNA synthesis and methylation, providing the non-reversible modification of 5,10-methylenetetrahydrofylate to 5-methyltetrahydrofylate [14]. Vitamin B12 oxidation, folate deficiency, and MTHFR inactivity are the main causes of elevated homocysteine levels and higher risk of thrombosis, cardiovascular disease, and carcinogenesis in animals and humans, for instance breast cancer, gastric cancer, and glioma [15].

Currently, there are two polymorphisms of the MTHFR gene: C677T and A1298C. MTHFR C677T polymorphism is responsible for enzyme inactivity and increased levels of homocysteine in heterozygotes and more in homozygotes. MTHFR C677T homozygous mutant individuals have elevated homocysteine levels, whereas heterozygous mutants have moderately higher ones compared to non-mutant individuals [16]. Rates of MTHFR C677T polymorphisms in homozygous status were also found to be increased in women with endometriosis and were associated with infertility. The role of the prothrombin G20210A mutation remains under investigation [17,18].

Recent research has demonstrated the association of MTHFR C677T polymorphism with an increased risk of tumors and particularly an increased risk of gastrointestinal and breast cancer, while MTHFR A1298C polymorphism has not been significantly associated with tumorigenesis. Nevertheless, MTHFR C677T and A1298C polymorphisms have been associated with cancer susceptibility, but the supporting evidence for this relationship has been found controversial [19].

Geographical distribution and ethnicity appear to be determinants of the association between MTFR polymorphisms and predisposition to malignancies. Indeed, studies in populations of different ethnicities showed or did not show an association of these polymorphisms with cancer types [20]. The combined genotypes of FVL and prothrombin G20210A, particularly in heterozygous carriers, have also been widely associated with higher risk of thromboembolic episodes while the risk of VTE increases with the number of prothrombotic alleles both in individuals with and without cancer, and the presence of thrombotic risk and cancer alleles leads to a 17-fold higher risk of VTE compared to individuals with a free history of cancer [21].

To our knowledge, there are no studies investigating the aforementioned gene mutations and in particular the combined genotypes and MTHFR mutations in combination with family history of cancer, deep vein thrombosis, and environmental exposure in Greece.

The aim of our study was to investigate the frequency of factor V Leiden (G1691A), factor II Prothrombin (G2021A), MTHFR C677T, MTHFR A1298, and combined polymorphisms in a Greek population by using real-time polymerase chain reaction methods and their possible association with family history of cancer and cardiovascular diseases (CVDs), as well as environmental epigenetic factors that might influence genotype expression.

## 2. Materials and Methods

This study was a cross-sectional single-arm. A residential area was randomly selected from the origin of Thessaloniki. According to the latest demographic data, this area consists of one thousand residents. After applying statistical power analysis (α = 0.05, beta = 0.2, power = 0.8) and the exclusion criteria, one hundred apparently healthy adults voluntarily participated in the study.

In particular, the total sample of one hundred apparently healthy adult blood donors (45 males and 55 females), aged 27.3 ± 9.1 years, were included in the study after providing their written consent prior to participation, in accordance with the Helsinki Declaration. Study protocol was approved by the Research Ethics Committee of the University of Western Macedonia. Exclusion criteria were non Caucasians, history of previous thrombosis, cancer, cardiovascular, neuromuscular disorders, autoimmune diseases, active systemic infection, psychiatric disorders, lactating and pregnancy, and blood relatives as our study was not family-based [22]. Participants were asked to complete a questionnaire about their demographic data, medical, and family history. Blood samples were collected in 2EDTA tubes and stored at −20 °C until processing.

DNA was isolated using the Quick-DNA Miniprep Kit (Zymo Research Corp, Irvine, CA, USA) and the frequency of the polymorphisms under study was determined by real-time PCR, using fluorescence probes with the Coagulation Panel Kit (Immunospark s.r.l., Rome, Italy).

### 2.1. DNA Isolation

DNA was isolated from the blood samples using the Quick-DNA™ Miniprep Kit. The isolation process resulted in DNA without the presence of RNA residues. The derivative of that procedure was then used to apply real-time PCR.

### 2.2. Real Time Polymerase Chain Reaction (RT-PCR)

Mutation control was performed by real-time PCR using fluorescence probes with the Coagulation Panel Kit (Immunospark s.r.l., Rome, Italy), including the use of primers MTHFR mutation probes, at position 677 for C>T and at position 1298 A>G, for detecting wild-type, mutant homozygotes, and heterozygous genotypes. The wild-type of the detector (WT) for MTHFR677 and 1298 were labelled with FAM fluorophore. The specific mutant sequence detectors were labelled with HEX fluorophore.

The real-time PCR method consisted of three steps: Step 1—DNA denaturation: high temperatures (94–95 °C) are required to achieve the breakdown of the double-stranded DNA into a single chain. Step 2—primer binding or hybridization: at this stage, the primers recognize and bind to the complementary sequences in the re-arranged DNA chains, and by the action of the enzyme DNA polymerase, deoxyribonucleotide bases are added to the third “end” of each primer, resulting in the synthesis of new DNA chains complementary to those of the original DNA molecule. The elongation phase took place at 72 °C, which is the optimum temperature for the polymerase to act.

Based on the analysis of the melting curve of the PCR products, gene polymorphisms were detected. The melting temperature of the probe for wild-type for factor V Leiden was 61 °C. The probe melting temperatures for prothrombin (G20210A), and MTHFR (C677T&A1298G), polymorphisms were 61 °C, too. Positive and negative controls were also included for each reaction.

## 3. Statistical Analysis

Statistical analysis was conducted using SPSS version 23 for Windows. Continuous variables were presented as means ± standard deviations (SD), and categorical ones as absolute and relative frequencies.

The expected frequencies were compared with the observed ones, using the Hardy–Weinberg Equilibrium chi square test. The frequency of each *single nucleotide polymorphism* (SNP) and mutation was analyzed using descriptive statistics and it was expressed as percentages. The frequency of the combined genotypes was tested using cross-tabulation analysis and expressed as percentages, also. The relation between two qualitative binary variables was checked by a χ^2^ test. All polymorphisms are presented in the genotypes analyzed: Wild Type homozygous (WT), Heterozygous (Het) and Mutated (Mut). Significance level was set at *p* ≤ 0.05.

## 4. Results

Descriptive characteristics of the participants are presented in Table 1.

**Figure 1:**
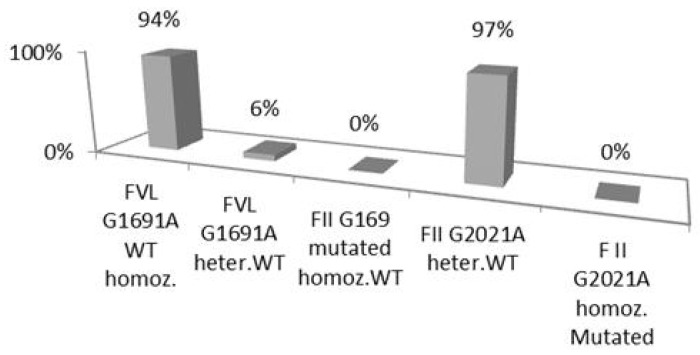
Genotypic frequencies of factor VL G1691A and factor II G2021A polymorphisms. WT hom. = wild-type homozygous; Heter. WT = heterozygous wild-type; Homoz. Mutated = homozygous mutated.

**Figure 2:**
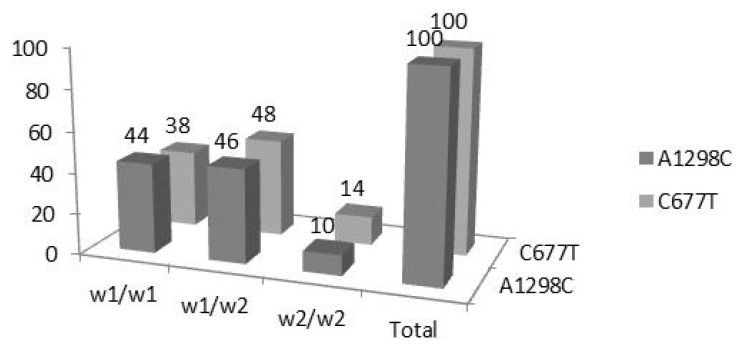
MTHFR genotyping frequencies expressed as % percentages. W1 = wild-type; W2 = Homozygous mutated.

The genotypic frequencies of the studied polymorphisms include the following: (a) factor VL G1691: 94/100 (94%) homozygous wild-type (WT), 6/100 (6%) heterozygous and 0/100 (0%), homozygous mutated (Mut); (b) factor II G2021A: 97/100 (97%) homozygous WT, 3/100 (3%), heterozygous and 0/100 (0%) homozygous Mut; (c) MTHFR C677T: 38/100 (38%) homozygous WT, 48/100 (48%), heterozygous, 14/100 (14%) homozygous Mut; (d) MTHFR A1298G: 44/100 (44%) homozygous WT, 46/100 (46%) heterozygous, 10/100 (10%) homozygous Mut (Figure 1 and Figure 2).

**Figure 3:**
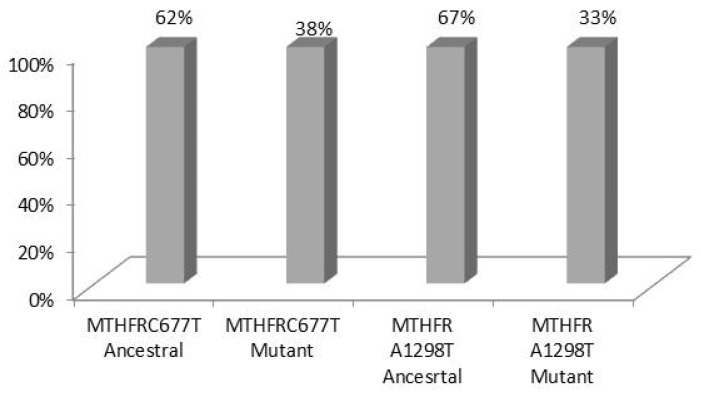
Allele frequencies of MTHFR C677T and MTHFR A1298C.

The allele frequencies (expressed as a % percentage), detected were for MTHFR C677T 62% for the ancestral allele, 38% for the mutant allele and for MTHFR A1298G 67% for the ancestral allele, and 33% for the mutant allele (Figure 3).

Carriers of two combined genotypes were the 52% of the sample size. Particularly, the combined MTHFR C677T and MTHFR A1298C genotype frequencies expressed as percentages include: WT/WT 8%, WT/Het 28%, WT/Mut 12%, Het/WT 16%, Het/Het 24%, Het/Mut 0%, Mut/WT 12%, Mut/Het 0% and Mut/Mut 0%, respectively (Table 2).

**Figure 4:**
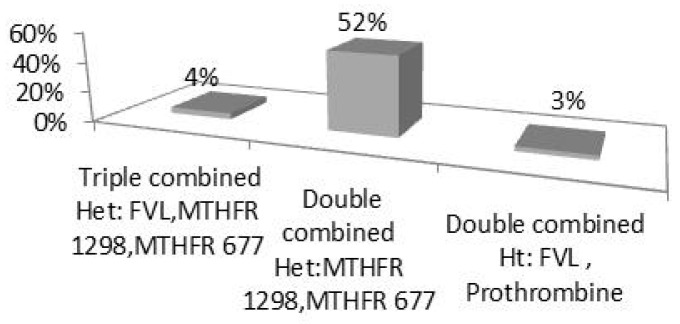
Frequencies of triple and double loci SNP genotypes.

In addition, a 4% frequency of a triple loci SNP genotype was found in association with the combination of FVL, MTHFR C677T, and MTHFR A1298C factors. A 3% frequency of a double loci SNP genotype was also observed regarding the combined FVL and PII genotypes (Figure 4).

Smoking and BMI status were significantly associated with combined MTHFR polymorphisms with the smokers and overweight subjects being more prone to have MTHFR combined genotypes (χ^2^ = 10, *df* = 2, *p* = 0.002 and χ^2^ = 14.6, *df* = 2, *p* = 0.001), respectively. Gender, age, and body weight were not significantly associated with MTHFR polymorphisms.

## 5. Discussion

Thrombosis, and more specifically venous thromboembolism, is a critical health disorder and the leading cause of mortality in young people with a higher prevalence globally. Studies have revealed that ethnicity, geographical area, and environment play a decisive and differential role in the distribution of genotypes and their phenotypic expression [23].

The observed frequencies of the FVL and FII prothrombin genotypes investigated in our study were similar to those reported for Caucasian populations. The frequency of polymorphism of factor VL G1691 homozygous wild-type was 94% and 6% of heterozygous type, while factor II G2021A was 97% homozygous wild-type and 3% heterozygous type. Mutant homozygosity was not present in any of the two above polymorphisms. The finding allele frequencies were as follows: factor VL: 97% for the ancestral allele and 3% for the mutated allele; factor II: 100% ancestral allele, 0% mutated allele.

In contrast, the study by Raptopoulou and colleagues, and the previously published data in Greek and in south Italian populations, showed a rate of about 2% to 2.2% in the incidence of the FVL homozygous mutant polymorphism [11,23,24,25]. On the other hand, a significantly higher prevalence of FVL (26%) was detected in Greek female subjects with pregnancy implications compared to those without any complications (8%) [17]. Regarding recurrent miscarriages, the evaluated frequencies of FVL and factor II in Greek women are also found significantly higher (e.g., 19% and 9%, respectively), but not with MTHFR 677C<T. MTHFR mutations possibly did not contribute to pregnancy implications [26].

Regarding the observed MTHFR C677T polymorphisms, our study found 38% homozygous WT, 48% heterozygous, and 14% in mutated condition which differed from those of the Greek Cypriot population in terms of 17.8% in the homozygous ones. The heterozygous state was similar [11,24]. Furthermore, the analysis also revealed that the distribution of MTHFR polymorphisms (heterozygous and mutated genotypes) differed significantly from the ones met in other studies on Greek population [24].

Homozygous mutant MTHFR 677C<T polymorphism prevalence was 14%, similar to the studied Greek population living in Athens [11], higher in Italian and Hispanic people, and lower in African Americans and in the African sub-Saharan area [27].

The frequency of 677C>T homozygosity was also higher in Hispanics and Italians. Nevertheless, MTHFR allele frequencies were not similar to the ones in other studies in Greece. Among Europeans, the homozygous allele frequency was found to be higher in Italians and lower in Germans. The rate of homozygosity in the population in Britain was approximately 13%. Among non-European Caucasians, the homozygous mutation frequencies varied from 10% to 14% in countries such as Canada, Australia, and Brazil [27].

The frequency of the MTHFR A1298C homozygous wild-type polymorphism was found in 44% of our series, and much higher than that reported in the Turkish population, which ranged from 10% to 13.3% [24]. Heterozygous wild-type mutation frequency was 46% and homozygous mutated carriers were 10% of the participants.

On the other hand, the distribution of MTHFR alleles were also significantly elevated. In particular, the frequency for wild-type MTHFR A1298C (C) alleles was 67% and for mutant alleles, (A) 33%. MTHFR C677T’s frequency was 62% for wild-type alleles (C) and 38% for mutant alleles (T). This indicates a relatively high prevalence of both mutations in the studied population. The strong correlation between A1298C and C677T frequencies might suggest a tendency for these mutations to occur together (compound heterozygosity), which could have more pronounced effects on MTHFR activity than either mutation alone. Investigating the frequency of MTHFR gene polymorphisms (A1298C and C677T), a high distribution of the 1298C allele (38.41%), has also been reported in Bosnia Herzegovina by Nefic and colleagues [28], while a lower and an absence of mutations were found in US populations [8].

The relatively high frequency of these mutations suggests they might confer some selective advantage, possibly related to folate metabolism, in different environmental conditions. These mutations may affect folate metabolism, potentially impacting processes like DNA synthesis and methylation. Reduced MTHFR activity can lead to elevated homocysteine levels [29], which is a risk factor for cardiovascular diseases, pregnancy implications [28], some cancer types, such as gastrointestinal and breast cancer [30], accompanied by comorbidities, like high blood pressure and arterial stiffness [19]. Breast cancer is a high incidence of breast tumors in women and is a leading cause of mortality. Gliomas are heterogeneous tumors and have high rates of both recurrence and mortality. Stomach tumors are the fifth more common tumor type with the third highest mortality rate. Scientific evidence has shown the effect of folate metabolism on DNA synthesis and methylation, which results in an elevated risk of different types of cancer when this pathway is disrupted. The concurrence of low folate levels in serum and MTHFR C677T mutation greatly increases the risk of developing malignancies [15].

Combined effects were also identified, as 4% of participants had three of the mutations studied, particularly for FV, MTHFR 677 C<T, and MTHFR 1298 A<C, and two combined gene mutations in more than half of the participants.

Environmental epigenomics plays a crucial role in gene expression. Lifestyle factors such as inadequate intake of folate and vitamin B12, smoking, being overweight and obesity, and ageing trigger the DNA damage process. Recent bodies of research have revealed associations between MTHFR C677T polymorphism, levels of homocysteine, smoking, and specific DNA methylation motives [31]. Our results highlighted a significant association among MTHFR combined genotypes, smoking, and weight status. The meta-analysis of twenty studies by Fu and colleagues, strengthens our findings by supporting the positive association of MTHFR C677T polymorphism with the risk of obesity and elevated serum homocysteine concentration, highlighting the importance of investigating the relationship between these factors and body weight status by means of body mass index [32].

Therefore, it is demonstrated that modifiable lifestyle factors, such as smoking and overeating, are associated with gene mutations, whereas previous evidence did not have a statistically significant association with weight [3], but it is consistent with the effect of tobacco use on MTHFR gene expression, as they have been widely identified as critical risk factors for thromboembolic events and cancer development [33].

Gender, age, and body weight were not significantly correlated with MTHFR polymorphisms in our sample. Regarding gender, the study by Zhi et al. indicated that MTHFR allele frequencies were significantly correlated between overweight and obese men and women. Being overweight and obesity have been detected based on BMI criteria rather than body weight [34]. In contrast, the study by Wang et al., with a sample size of 446 participants in China, has found a significant positive association with age-elevated homocysteine and MTHFR polymorphisms, more so in men than in women of reproductive age [34].

Interestingly, the observed frequencies of the MTHFR A1298C polymorphism and the combined mutations of FV, MTHFR 677C<T, and MTHFR 1298 A<C genotypes have not been widely investigated in Greece. Regarding MTFR, combined mutations should not be overlooked as they are mainly associated with tumors, infertility in males, endometriosis, and neurological disorders [7,18]. MTHFR gene polymorphisms, when associated to FVL heterozygous variants, increased the risk of arterial ischemic stroke in young Tunisian adults [7].

Importantly, while these mutations may be biologically significant, their clinical impact can vary considerably between individuals and populations. Factors such as diet (particularly folate intake), obesity, lifestyle and genetic agents all play a role in the way these mutations are manifested with health implications [35]. However, the results of studies are still controversial regarding the positive association between MTHFR polymorphisms and venous thrombosis. In particular, large-scale studies, such as the one conducted by the Copenhagen City Heart and Mega Studies, support the absence of an effect between MTHFR polymorphisms and cardiovascular disease and venous thromboembolism. Thus, MTHFR SNPs are not included in routine genetic testing for thrombophilia, and the detection of homocysteine levels is preferable [36].

Furthermore, our findings confirm the association between cancer susceptibility and MTHFR gene polymorphisms through family medical history, which was significantly associated with heterozygotes and mutant carriers of both polymorphisms.

The literature has controversially demonstrated the association between MTHFR C677T and MTHFR A1298C genotypes with breast cancer in female populations from different geographical regions such as China and Egypt, but underlining the increased risk of breast cancer in Caucasian women [18,28,31], while prothrombotic coagulation factors, FVL and FII, were not significantly associated with family history of colorectal cancer [32].

The strengths of our study were that we investigated combined genotypes in a sample of the resident population in Greece and found double and triple SNP genotype mutations, demonstrating the high importance of mutations of both MTHFR variants and their correlation to the confounding environmental factors (smoking and obesity). We also highlighted the importance of a detailed family medical history (CVDs and cancer), and the effect this might have on health disorders for descendants.

Limitations of this study include the small sample size and self-reported data on anthropometric indexes, medication, medical, and family history. Thus, our results cannot be generalized but could provide the stimuli for further investigation.

## 6. Conclusions

Our study provides crucial insights into the prevalence of factor V Leiden (G1691A), factor II prothrombin (G20210A), and MTHFR (C677T and A1298C) polymorphisms within a Greek demographic area. The frequencies of these polymorphisms generally align with those observed in Northern European populations, except for the MTHFR genotype with a higher distribution, underscoring the potential for geographical and ethnic influences on the prevalence of genetic mutations.

Notably, our findings extend beyond the association of these polymorphisms with thrombophilia. The data suggest a broader impact on health, including potential links to cardiovascular diseases and various forms of cancer, particularly when polymorphisms occur in combination. Such associations emphasize the significance of epigenetic factors and lifestyle choices, such as smoking and obesity, which may exacerbate the risk profiles associated with these genetic mutations.

This preliminary study highlights the necessity for further research to unravel the complex interactions between genetic polymorphisms and environmental factors.

Future studies should aim to expand sample sizes and include diverse Greek sub-populations to enhance the generalizability of the findings. Ultimately, this could lead to more refined strategies for early detection, prevention, and personalized treatment approaches, improving public health outcomes across susceptible populations.

Our research underscores the importance of integrating genetic screening into public health strategies, particularly for populations at heightened risk of thrombosis and related complications. The interplay of genetic predispositions and lifestyle factors offers a valuable perspective for developing comprehensive health management plans that cater to the specific needs of individuals based on their genetic and environmental risk factors.

Gene polymorphisms are not only involved in thrombosis but are associated with many other health issues. The distribution of FVL, F II, and MTHFR gene mutations was shown to follow the distribution of Northern Europe and differs from other regions. Combined mutations and epigenetic factors should attract more research to create better prediction patterns and strategies in primary preventive health.

## Figures and Tables

**Table 1:** Demographic characteristics.

	Mean ± SD	N (%)
Age (years)	27 ±9	
BMI (kgr/m^2^)	25 ± 5	
Body Weight (kgr)	75 ± 18	
Smokers		64 (64%)
Males		45 (45%)
Females		55 (55%)
CVD Fam. History		20 (20%)
Cancer Fam. history		15 (15%)
Contraceptives		2 (2%)
Folic acid sump.		8 (8%)
Heterozygous β Thalassemia		8 (8%)

SD = standard deviation; N = number of participants; CVD Fam. History = CVD family history; Cancer Fam. History: cancer family history; Folic acid sump.: folic acid supplementation.

**Table 2:** MTFHR combined genotypes.

MTHFR 677C<T/MTFR1298 A/C	Frequencies%
WT/WT homoz.	8%
WT/heter.	28%
WT/Mut.	12%
Heter./WT	16%
Heter./Heter.	24%
Heter.Mut.	0%
Mut/WT	12%
Mut/Heter	0%
Mut./Mut.	0%

WT = wild--type; Heter. = heterozygous; Mut. = mutated.

## Data Availability

Data are contained within the article.
